# Reply to: What evidence is required to justify the NHS Health Check programme?

**DOI:** 10.1186/s12916-025-04080-4

**Published:** 2025-04-25

**Authors:** Zahra Raisi-Estabragh, Stefan Neubauer, Thomas E. Nichols, John Robson

**Affiliations:** 1 William Harvey Research Institute, NIHR Barts Biomedical Research Centre, Queen Mary University of London, Charterhouse Square, London, EC1M 6BQ UK; 2 Barts Heart Centre, St Bartholomew’s Hospital, Barts Health NHS Trust, West Smithfield, London, EC1 A 7BE UK; 3 Division of Cardiovascular Medicine, Radcliffe Department of Medicine, University of Oxford, National Institute for Health Research Oxford Biomedical Research Centre, Oxford University Hospitals NHS Foundation Trust, Oxford, UK; 4 Big Data Institute, Li Ka Shing Centre for Health Information and Discovery, Nuffield Department of Population Health, University of Oxford, Oxford, UK; 5 Wolfson Institute of Population Health Queen, Queen Mary University of London, Charterhouse Square, London, UK

**Keywords:** Population health, Preventive medicine, Cardiovascular disease, National health service, UK biobank

## Abstract

Medications that reduce blood pressure and cholesterol are among the most cost-effective healthcare interventions available, but their delivery remains suboptimal. In 2009, the National Health Service (NHS) Health Check was introduced to increase the detection and treatment of major cardiovascular risk factors in people aged 40–74 years. In a prospective observational study using the UK Biobank, we compared health outcomes between NHS Health Check attenders and matched non-attenders. Attenders exhibited higher early rates of new hypertension, hyperlipidaemia, and chronic kidney disease, followed by significantly lower long-term multi-system disease and mortality. A recent critique of our work raises questions regarding observational design limitations, self-selection bias, and discrepancies with randomised controlled trials (RCTs). However, RCTs face ethical and feasibility challenges in large-scale public health interventions and are not immune to self-selection. Furthermore, the argument that self-selection explains our findings is inconsistent with our results. If healthier individuals were disproportionately attending NHS Health Checks, we would expect lower risk across all outcomes. However, we observed an initial increase in new diagnoses suggesting that NHS Health Checks are detecting pre-existing conditions earlier rather than merely attracting healthier individuals. Additionally, the cited RCTs predate modern antihypertensive and statin treatments, and considered heterogenous non-validated interventions. In summary, this critique relies on selective citation of outdated and inappropriate RCTs, an overstatement of selection bias, and an underappreciation of the role of observational research in shaping public health improvements. Our findings indicate that NHS Health Checks contribute to the prevention of multi-system morbidity and mortality, warranting continued investment.

Cardiovascular disease (CVD) remains the most common cause of death worldwide, with a recent plateauing of declining mortality trends. Medications that lower blood pressure and serum cholesterol are among the most cost-effective preventive interventions [1, 2], but are not being delivered optimally.

In 2009, the National Health Service (NHS) Health Check was established in England, aiming to increase the detection and treatment of major cardiovascular risk factors through a national rolling five-yearly programme, in people aged 40–74 years who do not already have established CVD, diabetes, chronic kidney disease (CKD), or hypertension [3]. The NHS Health Check comprises a standardised and validated clinical consultation in primary care including blood pressure measurement (which includes pulse regularity), a blood test for measurement of serum lipids and where appropriate glycaemic control. Advice on smoking cessation and high alcohol consumption is offered, with referral for management of detected hypertension, statins for CVD risk or diabetes, and tests for CKD and atrial fibrillation where indicated.

In a recent prospective observational analysis, we examined disease and mortality outcomes in UK Biobank participants who had completed an NHS Health Check, compared to matched non-attenders [4]. In the early period after the NHS Health Check, attenders had higher rates of new diagnoses of hypertension, high cholesterol, and CKD. While, in the longer-term, we observed lower rates of multi-system disease and mortality events in attenders, compared to non-attenders.

We read with interest, the commentary by Jørgensen et al. [5], which critiques our study on several grounds, including the reliance on observational data, potential confounding by self-selection, disagreement with randomized controlled trial (RCT) findings, and lack of discussion on harms.

We agree that evaluating the health benefits of programmes designed to identify earlier CVD risk and management is challenging. Individuals who take-up such interventions are in general healthier, more affluent, and have higher educational attainment, than those who do not, with self-selection leading to lower adverse outcomes in attenders due to confounding [6].

In our UK Biobank study, attenders and non-attenders were matched on an extensive range of sociodemographic, lifestyle, and baseline morbidity factors [4]. The argument that self-selection entirely explains our findings is inconsistent with our results. If healthier individuals disproportionately attended NHS Health Checks, this would tend towards *lower* yield of new diagnoses than in non-attenders. However, we observed an initial increase in new diagnoses of major metabolic conditions shortly after attendance, indicating that NHS Health Checks are detecting previously undiagnosed pre-existing conditions earlier. Our findings corroborate similar study reports of increased diagnoses of diabetes, hypertension, and CKD after the NHS Health Check [7], and extend these to additionally demonstrate associations with significant reductions in long-term multi-system disease and mortality events.

While RCTs are the gold standard for establishing causality in many contexts, they are not always feasible for large-scale population health interventions. Observational studies have long informed public health policy. For example, the association of smoking and lung cancer is based almost entirely on observational data.

Furthermore, the issue of self-selection for health interventions may also hamper RCTs—making trial designs challenging. For example, the Inter99 Copenhagen trial showed that among people invited for a health check, mortality from unrelated causes (e.g. road traffic accidents) was substantially lower among attenders than non-attenders [8]. High quality RCTs at scale with therapeutic intervention in routine primary care settings that include cardiovascular mortality as outcomes are difficult to achieve, even in experimental settings.

Jørgensen et al. cite a meta-analysis of 15 historic RCTs, dated 1963–1999, reporting no mortality benefit of health checks [9]. The interventions used were highly heterogeneous and lacked validation – including general health consultations, blood tests, pelvic examinations, chest radiographs, mammography, and sigmoidoscopy alongside a range of behavioural interventions in a wide variety of clinical and community settings. Outcomes were similarly diverse, largely unvalidated, generally lacking in power and often incomplete. These trials differed fundamentally from the NHS Health Check in aims, interventional design, implementation, scale and outcomes.

Two further studies were cited as evidence against health checks: The Danish DANCAVAS trial [10], which focused on screening with cardiac CT scans and biomarkers rather than the structured, risk-based approach of NHS Health Checks; and the ADDITION-Cambridge trial [11], which was designed for diabetes screening alone and does not reflect the broader cardiovascular prevention in NHS Health Checks.

Importantly, most of the cited studies took place long before effective treatment with antihypertensives and statins were established in routine clinical practice in the late-1990s. In the UK, it was not until 2004 that national guidance on hypertension treatment was published, and 2008 when statin treatment for primary prevention based on multifactorial risk estimation was introducted [5]. It is thus inappropriate and potentially misleading to extrapolate these earlier heterogenous studies to the NHS Health Check, which started in 2009.

The further suggestion from Jørgensen et al. is that the NHS Health Checks causes harm by diverting resources from more critical services. Early disease detection and management supports cost-effective management, with prevention of costly complications such as ischaemic heart disease, stroke, heart failure, and renal failure. National Institute for Health and Care Excellence (NICE) evaluations recommend treatment of hypertension and use of statins as cost-effective [1, 2]. The assertion that NHS Health Checks are a net burden on the health service is a claim without substance.

In summary, Jørgensen et al. raise important questions about the evidence base for NHS Health Checks, but their critique relies on selective citation of outdated and inappropriate RCTs, an overstatement of selection bias, and an underappreciation of the role of observational data in shaping health policy and public health improvement. Our findings suggest that NHS Health Checks contribute to the prevention of cardiovascular and multi-system morbidity and mortality, warranting continued investment and further research into their optimisation.

## Data Availability

No datasets were generated or analysed during the current study.

## Abbreviations

CKD: Chronic kidney disease
CVD: Cardiovascular disease
NHS: National Health Service
NICE: National Institute for Health and Care Excellence
RCT: Randomized controlled trial
